# Thrombotic Microangiopathy in Pregnancy: Current Understanding and Management Strategies

**DOI:** 10.1016/j.ekir.2024.05.016

**Published:** 2024-05-22

**Authors:** Manuel Urra, Shannon Lyons, Corina Gabriela Teodosiu, Richard Burwick, Anuja Java

**Affiliations:** 1 Department of Renal Medicine and Hypertension, University of Colorado Anschutz Medical Campus, Aurora, Colorado, USA; 2 Carol Davila University of Medicine and Pharmacy, Bucureşti, Romania; 3 Maternal Fetal Medicine, San Gabriel Valley Perinatal Medical Group, Pomona Valley Hospital Medical Center, Pomona, California, USA; 4 Division of Nephrology, Department of Medicine, Washington University School of Medicine, St. Louis, Missouri, USA

**Keywords:** complement, microangiopathic hemolytic anemia, pregnancy, thrombocytopenia, thrombotic microangiopathy

## Abstract

Thrombotic microangiopathy (TMA) represents a heterogeneous group of disorders characterized by microvascular thrombosis and end-organ damage. Pregnancy-associated thrombotic microangiopathy (p-TMA) has emerged as a distinct clinical entity with unique diagnostic challenges. Identifying the specific form of p-TMA is critical for appropriate and timely management. This review offers a comprehensive overview of the various forms of thrombotic microangiopathies associated with pregnancy, highlighting our current understanding of their pathophysiology and the evolving landscape of diagnosis and treatment for each.

TMA is a clinicopathologic entity typically characterized by microangiopathic hemolytic anemia (hemoglobin level <10 g/dl, a lactate dehydrogenase [LDH] serum level >1.5 times the upper limit of normal, undetectable serum haptoglobin, negative direct erythrocyte antiglobulin test, and presence of schistocytes on peripheral smear), thrombocytopenia (platelet count <150 × 10^9^/l), and end-organ damage with a predilection for the renal, cardiac, and nervous systems.^1^, ^2^, ^3^, ^4^, ^5^ The clinical manifestations occur because of endothelial injury and microthrombi formation in small blood vessels, leading to ischemic organ injury.^6^ TMA can also exist with less extreme values and incomplete presentations, for example, thrombocytopenia can be mild or absent in a subset of patients. Renal presentation can vary across patients, presenting as renal-limited TMA (without any hematological manifestations), often associated with autoimmune diseases, drugs, or solid organ transplantation.^6^^,^^7^ Evaluation of a TMA should include a complete blood count, comprehensive metabolic profile, urinalysis and urine protein quantification, reticulocyte count, LDH, haptoglobin, direct Coombs test, coagulation profile, and a peripheral smear.

A kidney biopsy (when feasible) may be performed to confirm the diagnosis, evaluate the extent of organ damage and to rule out other etiologies of kidney failure. Pathologically, TMA is characterized on light microscopy by the presence of fibrin thrombi within the glomeruli. However, in some biopsies, overt thrombosis is not seen. Nonthrombotic features include endothelial swelling and mesangiolysis (in active lesions), double contours of the glomerular basement membrane (in chronic lesions) as well as accumulation of fluffy material in the subendothelium. Intramural fibrin deposition, myxoid intimal thickening, and concentric myo-intimal proliferation (onion-skinning) may occur in arteries and arterioles. Cases with absence of overt thrombosis can be best described as microangiopathy without thrombosis, which may represent either a sampling issue or the chronic phase of injury.^8^

There are multiple etiologies of a TMA, with pregnancy and the postpartum period being a high-risk time for TMA development .^9^, ^10^, ^11^, ^12^ Four major forms of p-TMA include thrombotic thrombocytopenic purpura (TTP),^13^ Hemolysis, Elevated Liver enzymes, and Low Platelet count (HELLP) syndrome often seen in conjunction with preeclampsia with severe features (PE-SF),^14^ complement-mediated thrombotic microangiopathy (CM-TMA) or atypical hemolytic uremic syndrome (aHUS)^3^ and TMA associated with antiphospholipid syndrome (APS).^15^^,^^16^ In addition, there can be other pregnancy-related complications that can present with features of a TMA, including sepsis, placental abruption and postpartum hemorrhage.^9^ In pregnancy, a diagnosis of TMA results in an ∼4.5 times higher risk of mortality compared with pregnant patients without TMA.^17^ Morbidity is also high with 81% of patients requiring dialysis and 46% progressing to end-stage kidney disease (ESKD).^7^ It can be challenging to differentiate between the various etiologies of p-TMA given the clinical and laboratory similarities, however, timely diagnosis is key for appropriate management and to prevent ESKD and other associated complications including myocardial ischemia, seizures, strokes, and even death.^1^^,^^17^

In this review we seek to define the prevalence, pathophysiology, diagnostic evaluation, treatment, and long-term outcomes of the 4 major causes of p-TMA mentioned above as well as highlight the latest developments in the field.

### TTP

TTP is a life-threatening condition that presents as a TMA. Pregnancy is a well-described precipitant for the development of this disease. TTP occurs at a rate of ∼2 in 100,000 pregnancies.^18^ Overall, it is estimated that approximately half of all acute TTP episodes occur in women of childbearing age and that pregnancy-associated TTP accounts for 12% to 25% of adult-onset TTP cases.^19^ TTP may be congenital (cTTP) or acquired/immune (iTTP).

### Etiology/Pathophysiology

The underlying mechanism of TTP is a deficiency in a key enzyme, ADAMTS13 (a disintegrin and metalloproteinase with a thrombospondin type 1 motif member 13) also known as von Willebrand factor (VWF)–cleaving protease. ADAMTS13 is a metalloproteinase that cleaves ultra large multimers of VWF^20^ (Figure 1a). ADAMTS13 deficiency leads to uncleaved ultra large VWF multimers which serve as a scaffolding for platelet adhesion and aggregation with resultant development of microthrombi formation. Microvascular thrombosis leads to the development of microangiopathic hemolytic anemia, thrombocytopenia, and tissue ischemia.^21^ The etiology of ADAMTS13 deficiency in cTTP is a recessive mutation (homozygous or compound heterozygous) in the ADAMTS13 gene, whereas in iTTP, acquired autoantibodies inhibit the proteolytic activity of the metalloproteinase, as demonstrated by positive anti-ADAMTS13 IgG in ∼75% of iTTP.^22^

**Figure 1:**
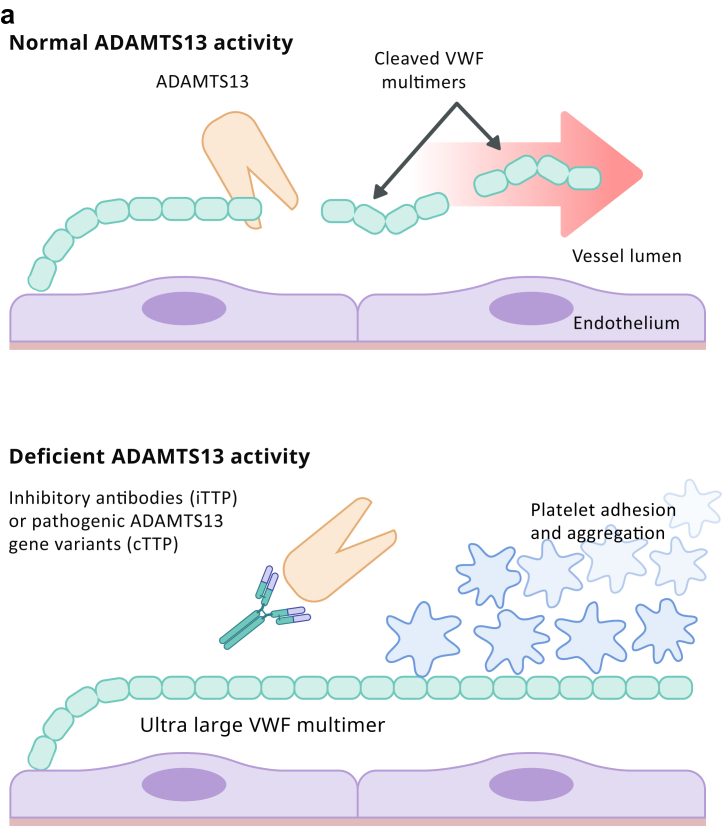

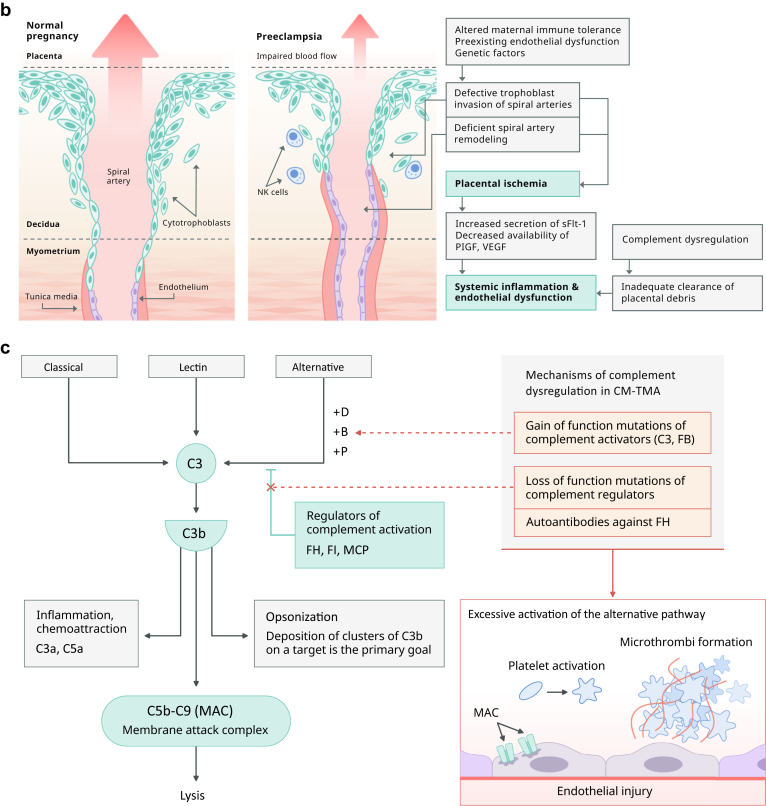

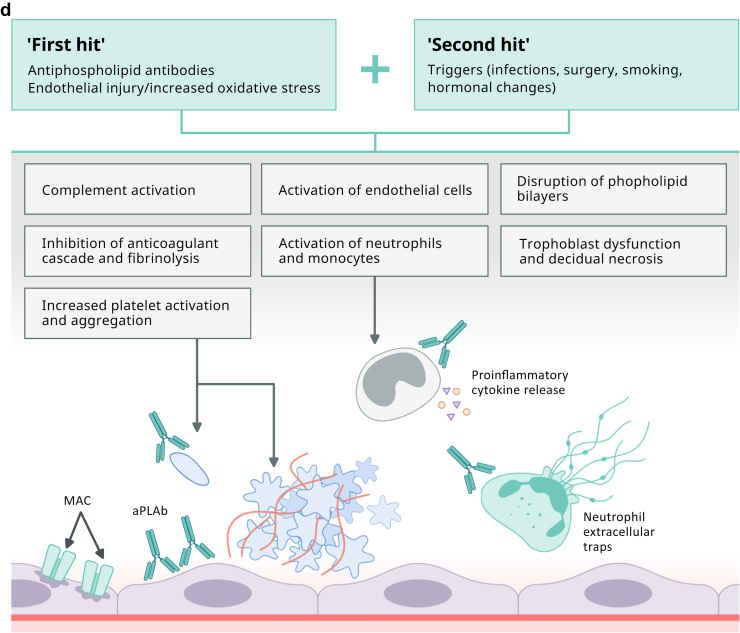
(a) Pathogenesis of Thrombotic Thrombocytopenic Purpura (TTP). ADAMTS13 is a plasma protease that cleaves ultra large VWF multimers into smaller multimers. Deficient enzymatic activity (caused by either inhibitory antibodies or pathogenic gene variants) leads to accumulation of ultra large multimers on the endothelial surface, providing a scaffolding for platelets to attach. ADAMTS13, a disintegrin and metalloproteinase with a thrombospondin type 1 motif, member 13; cTTP, congenital TTP; iTTP, immune TTP; VWF, von Willebrand Factor. (b) Pathogenic mechanisms involved in HELLP sSyndrome. Defects in spiral artery remodeling and trophoblast invasion result in abnormal placentation and placental ischemia. The subsequent release of inflammatory mediators, antiangiogenic factors and placental debris into the maternal bloodstream drive the systemic inflammation and endothelial dysfunction responsible for the clinical features of HELLP. HELLP, Hemolysis with Elevated Liver enzymes and Low Platelets; NK cells, natural killer cells; PIGF, placental growth factor; sFlt-1, soluble fms-like tyrosine kinase-1; VEGF, vascular endothelial growth factor. (c) Complement pathway and mechanisms of dysregulation in Complement-mediated TMA (CM-TMA). Complement gene mutations or autoantibodies against FH cause excessive AP activation followed by MAC-induced endothelial cell injury and microthrombi formation. AP, alternative pathway; FB, factor B; FH, factor H; FI, factor I; MAC, membrane attack complex. MCP, membrane cofactor protein. (d) Pathogenic mechanisms contributing to Antiphospholipid syndrome (APS) associated TMA. In the “two-hit” model of APS, a trigger event is required to cause manifestations of the disease in patients with preexisting aPL antibodies. Multiple mechanisms contribute to the proinflammatory and procoagulant state that drives pregnancy morbidity in APS syndrome associated TMA. aPLAb, antiphospholipid antibodies.

Pregnancy increases the risk for the development of TTP because of several factors: VWF levels increase as early as the first trimester of pregnancy, and supranormal levels persist through 6 weeks postpartum, leading to a decrease in ADAMTS13 levels because of consumption.^23^ This normal physiologic alteration of pregnancy may unmask cTTP by further depleting already low ADAMTS13 levels. Thus, patients with cTTP may not manifest any clinically significant symptoms until the physiologic stress of pregnancy is underway. The incidence of cTTP is therefore higher in pregnant individuals when compared to the general population. Two distinct TTP cohorts^24^^,^^25^ have shown the incidence of cTTP to be between 24% and 66% of all pregnancy related TTP cases, compared with <5% of nonpregnancy related TTP.

### Clinical Features and Diagnosis

Although pregnancy-associated TTP can occur anytime during gestation, it is most common in the third trimester and postpartum period. However, if a TMA is present in the first trimester, TTP is still the most likely diagnosis. Clinical features may include fever, neurologic manifestations, and renal injury. TTP has a particularly stronger predilection for neurologic end organ damage manifesting as encephalopathy, seizures, and cerebrovascular accidents. Severe thrombocytopenia (<30,000/μl) is often present and is more common in TTP, whereas renal injury is usually milder compared with other TMAs.^26^

The diagnosis of iTTP is confirmed by an ADAMTS13 activity level <10% and the presence of ADAMTS13 IgG (Figure 2). cTTP is diagnosed when ADAMTS13 activity level is <10% in the absence of ADAMTS13 IgG and confirmed by mutational analyses. Genetic testing should be conducted to establish the presence of a homozygous or a compound heterozygous mutation in the ADAMTS13 gene.^27^ However, if acute TTP is suspected based on clinical and laboratory features, treatment should not be delayed while waiting for ADAMTS13 results. PLASMIC score may be used to discern between high-risk (score >5) versus intermediate-low risk of ADAMTS13 deficiency and can help to differentiate TTP from other TMAs (although these scores were developed outside the pregnancy setting).^28^ Overall, a patient with p-TMA who presents with neurological manifestations and severe thrombocytopenia with only mild kidney involvement is most likely to have TTP.

**Figure 2:**
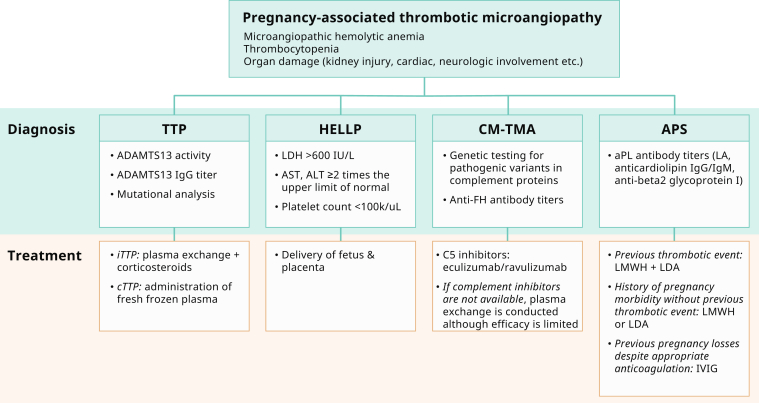
Diagnosis and treatment by type of pregnancy-associated TMA. ADAMTS13, a disintegrin and metalloproteinase with a thrombospondin type 1 motif, member 13; aPL, antiphospholipid; APS, antiphospholipid syndrome; ALT, alanine aminotransferase; AST, aspartate aminotransferase; CM-TMA, complement-mediated thrombotic microangiopathy; cTTP, congenital TTP; FH, factor H; HELLP, Hemolysis, Elevated Liver enzymes, Low Platelet count syndrome; IVIG, intravenous immunoglobulin; iTTP, immune TTP; LA, lupus anticoagulant; LDA, low-dose aspirin; LDH, lactate dehydrogenase; LMWH, low molecular weight heparin; TTP, thrombotic thrombocytopenic purpura.

### Treatment and Prognosis

Treatment should be promptly initiated while awaiting diagnostic work up results (Figure 2). The management of iTTP in pregnancy involves daily plasma exchange (PLEX); 1 to 1.5 plasma volume using fresh frozen plasma and corticosteroids (oral prednisone 1 mg/kg per day). PLEX is an extracorporeal procedure with the aim of removing patient’s plasma and replacing it with fresh frozen plasma, effectively removing autoantibodies and ultra large VWF multimers while replacing functional ADAMTS13 and fresh plasma components. The optimal duration of PE is unknown, but it is recommended to continue PE for a minimum of 2 days after remission is obtained, as defined by normalization of neurological status, platelet count and LDH as well as rising hemoglobin levels. It may also be acceptable to taper the frequency of exchanges rather than stopping abruptly to minimize the risk of relapse.^4^^,^^29^ Before the use of PLEX, survival from TTP was 10%. With prompt initiation of PLEX, the average survival rate after the first episode of TTP is 80% to 90%.^30^ Other immunosuppressants such as azathioprine and calcineurin inhibitors have also been used to treat iTTP.^31^, ^32^, ^33^, ^34^ There is limited data on the use of rituximab during pregnancy since the drug crosses the placenta, especially during the third trimester.^35^ However, more recently, use of caplacizumab, a monoclonal antibody against VWF which blocks the adhesion of VWF multimers to platelets was reported in 2 pregnant individuals^36^^,^^37^ (See Table 1). In the first report, the authors describe a patient with refractory iTTP at 18 weeks gestation. The patient was treated off-label with caplacizumab, which led to platelet count normalization within 3 days. However, over the next few weeks, the pregnancy worsened with development of preeclampsia, severe growth restriction, oligohydramnios, and placental hydrops. Termination of pregnancy was recommended at 21 weeks, and fetocide was performed. Transplacental transfer of caplacizumab was documented with drug levels identified in amniotic fluid and fetal blood. In the second case, the authors reported a pregnant woman at 28 weeks gestation with refractory iTTP (exacerbation of disease despite daily PLEX and steroids).^37^ Addition of caplacizumab led to rapid resolution of thrombocytopenia. At 33 weeks gestation, caplacizumab was held due to planned delivery secondary to fetal heart rate decelerations. The neonatal course was unremarkable aside from issues related to prematurity, and the neonate was discharged on day of life 23. In both cases, caplacizumab achieved control of platelet count in a refractory disease, though adverse pregnancy outcomes could not be prevented. Given that these are the only 2 cases reported in the literature so far, use of the drug should be based on shared-decision making and should involve careful risk-benefit discussion with the patient. It has also been proposed that caplacizumab may theoretically increase risk of maternal and neonatal bleeding, therefore its use should be reserved for refractory cases. At the same time, delaying the introduction of caplacizumab may expose both the mother and the fetus to a fatal outcome. These observations highlight the need for more data and formal studies to establish the safety, efficacy, and timing of use of caplacizumab for the treatment of iTTP during pregnancy. Additional agents, such as cyclophosphamide and splenectomy are not recommended in pregnancy.

**Table 1:** Summary of What’s New in TMA in Pregnancy

1) Caplacizumab is a humanized monoclonal antibody that works by binding to the A1 domain of VWF and blocks its interaction with platelets via the platelet glycoprotein Ib receptor, thereby preventing the formation of microthrombi. At the time of the report from the international working group on TMA in pregnancy in 2020, there was no safety data of caplacizumab in pregnant patients. Since then, use of caplacizumab has been reported in two iTTP cases in pregnancy with details outlined in the TTP section above.^36^^,^^37^ More data is needed to fully establish the safety and efficacy of caplacizumab in pregnancy.
2) The therapeutic arsenal of cTTP may also soon be expanded with the availability of recombinant ADAMTS13. A pregnant patient was successfully treated with the recombinant form for cTTP that was not controlled with plasma infusions.^38^ Since the publication of this case report, results of a phase 3, open-label trial involving 48 patients randomly assigned to recombinant ADAMTS13 or standard therapy, have been published.^39^ During prophylaxis with recombinant ADAMTS13 in patients with cTTP, ADAMTS13 activity reached approximately 100% of normal levels and no acute TTP event occurred. This therapy opens a promising avenue for the treatment of pregnant women with cTTP.
3) The Food and Drug Administration (FDA) has cleared the blood-based biomarkers (sFlt1/PIGF) to aid in the risk assessment and clinical management of hypertensive disorders of pregnancy. Based on the recent PRAECIS study, an sFlt-1:PlGF ratio ≥40 is predictive of severe PE and adverse outcomes within the ensuing 2 weeks.^59^ Details of the PRAECIS study and the Latest Clinical Practice Update recommendations from ACOG are outlined in the HELLP section.^59^^,^^60^ Further research is needed to determine if the sFlt-1/PlGF ratio can effectively differentiate PE from other pregnancy TMA disorders.
4) A recent study demonstrated that HELLP can potentially be differentiated from CM-TMA by the combination of serum creatinine and LDH. A serum creatinine greater than 1.9 mg/dL and LDH greater than 600 U/L at 72hr postpartum has been shown to be more than 95% specific for the diagnosis of CM-TMA and may help differentiate HELLP from CM-TMA. Details of the study can be found under the HELLP section above. Until more advanced testing modalities are available, serum creatinine and LDH may be used to quickly identify women with suspected CM-TMA in the postpartum period.^61^
5) A newer long-acting C5 inhibitor, ravulizumab is now approved for CM-TMA.^82^ It’s use in postpartum CM-TMA has been reported in 8 patients with complete response in 7 of 8 patients in 31 days. Breastfeeding is not recommended with ravulizumab until safety data is available.
6) In addition to eculizumab and rituximab mentioned above, a number of new biologic agents are in trials for APS.^135^ These include obinutuzumab (type II anti-CD20 monoclonal antibody), belimumab [B cell antibody targeting the soluble circulating B cell activating factor (BAFF); NCT05020782], daratamumab (anti CD38 antibody), adalimumab (Anti-TNF-α therapy), certolizumab (Anti-TNF-α therapy; NCT03152058) and zanubrutinib (Bruton kinase inhibitor; NCT05199909). Of these, belimumab, adalimumab, and certolizumab use has been reported for severe and refractory disease in pregnancy although limited by small sample sizes (details under the APS section). Future trials are awaited to inform us about the use of these promising agents for personalized medicine in pregnancy.

Treatment for cTTP centers around administration of fresh frozen plasma which replaces deficient levels of ADAMTS13 and in more severe cases, PLEX may serve to remove VWF multimers. If a diagnosis of cTTP is known before pregnancy, prophylactic fresh frozen plasma administration (typically 10–15 ml/kg plasma every 2 weeks in the first trimester with potential need to increase frequency to weekly or every other day in second or third trimesters) should be initiated immediately after pregnancy and continued throughout pregnancy and likely until 4 to 6 weeks postpartum period with the goal to achieve normalization of platelet count, LDH and absence of symptoms as well as maintenance of ADAMTS13 activity levels >20% to 25%.^4^^,^^31^ The therapeutic arsenal of cTTP may soon be expanded with the availability of recombinant ADAMTS13 (See Table 1). In a recent report, a 27-year-old pregnant patient with cTTP (confirmed by an ADAMTS13 activity, 3.9% and presence of biallelic ADAMTS13 mutations: c.2017A→T (p.I673F) in exon 17 and c.3178C→T [p.R1060W] in exon 24), who was refractory to plasma therapy, was treated with recombinant ADAMTS13 at 32 weeks gestation, with weekly injections of 40 IU per kilogram.^38^ The patient’s platelet count improved rapidly and she delivered a small-for-gestational-age baby (1865 grams) at 37 weeks. The neonate had no reported complications and was discharged on day of life 7. Since the publication of this case report, investigators for the cTTP study have reported results of a phase 3, open-label, crossover trial.^39^ Forty-eight patients were randomly assigned in a 1:1 ratio to 2- and 6-month periods of prophylaxis with recombinant ADAMTS13 (40 IU per kilogram of body weight, administered intravenously) or standard therapy, followed by the alternate treatment; thereafter, all the patients received recombinant ADAMTS13 for an additional 6 months. Thirty-two patients completed the trial. No acute TTP event occurred during prophylaxis with recombinant ADAMTS13, whereas 1 patient had an acute TTP event during prophylaxis with standard therapy. The mean maximum ADAMTS13 activity after recombinant treatment was 101%, as compared with 19% after standard therapy. Adverse events were generally mild or moderate in severity. This recombinant therapy opens a promising avenue for the treatment of pregnant women with cTTP.

Delayed or absent treatment of either form of TTP may result in placental infarction and fetal death. Maternal complications can include stroke, myocardial infarction, acute kidney injury, disseminated intravascular coagulation or other complications of severe thrombocytopenia. Women are also at increased risk for depression and,^40^ cognitive impairment with a substantial proportion of patients demonstrating silent cerebral infarctions on MRI.^41^ These patients often develop new onset hypertension^42^ and are at risk for relapsing TTP.

### HELLP Syndrome

The most common cause of p-TMA is HELLP syndrome, often occurring in the context of PE-SF. HELLP syndrome can occur in ∼0.5% to 1% of all pregnancies with more than two thirds of cases occurring in the third trimester, and some occurring in the immediate postpartum period.^43^ The maternal mortality rate of HELLP syndrome is estimated at 1.1% and perinatal death rate reported at 7% to 34% of cases.^44^^,^^45^

### Etiology/Pathophysiology

The exact pathophysiologic mechanism for the development of HELLP is not fully understood. It is however generally accepted that abnormal placentation plays a significant role in its development (Figure 1b). Pregnancy represents an allogeneic-type mismatch between the fetus and the maternal immune system. Since the fetus encounters maternal immunocompetent cells during trophoblast invasion, healthy placentation depends on a collaboration between efficient trophoblast invasion and the mother’s immune system. During trophoblast invasion, many immune cells infiltrate the decidua, allowing them to reach the endometrium and spiral arteries.

In healthy pregnancies, nonrecognition of these trophoblasts by maternal immune cells promotes maternal-fetal tolerance. However, an overactive maternal immune system can negatively impact this process. It can activate programmed death of trophoblasts, known as apoptosis, significantly disrupting their migration and placental vascularization. This in turn can lead to placental ischemia and release of inflammatory mediators by the placenta. Increasing evidence suggests that an overactive maternal immune system in pregnancy could occur because of a dysregulated complement system. This has been supported by reports in the literature demonstrating an increase in markers of complement activation (C5a and C5b-9) in PE/HELLP. Additionally, it has been shown that C5b-9 deposition on endothelial cells was increased following exposure to plasma from women with PE/HELLP syndrome.^46^

The involvement of complement in adaptive immune response as well as its interaction with natural killer cells are potential ways in which homeostatic balance and control over invasive placental trophoblast cells is achieved. Moreover, during pregnancy, complement helps clear placental fragments that enter the maternal circulation from trophoblast turn-over. Improper clearance of such components, driven by an inadequately regulated complement system, may cause deposition of debris in tissues and vascular walls, leading to an overly exuberant inflammatory response, resulting in endothelial injury and placental dysfunction.^47^ A dysregulated complement system can occur due to genetic mutations in complement proteins (factor H, factor I, membrane cofactor protein, or C3) as identified in up to 45% patients with HELLP (range 5%–45% across numerous studies).^48^^,^^49^

Complement activation can also occur as a result of imbalance in the production between antiangiogenic (soluble fms-like tyrosine kinase-1 [sFlt-1]) and pro-angiogenic factors (vascular endothelial growth factor and placental growth factor [PlGF]) by placenta. In vitro studies have shown that vascular endothelial growth factor induces complement Factor H synthesis by endothelial cells and protects the endothelium through modulation of local complement proteins. sFlt-1 is an endogenous inhibitor of vascular endothelial growth factor and can thereby promote aberrant complement activation and complement-mediated placental damage through vascular endothelial growth factor inhibition at the maternal-fetal interface. Increased secretion of sFlt-1 that binds PlGF can also result in impaired placental blood flow, oxygenation and poor spiral artery remodeling further contributing to placental inflammation and endothelial dysfunction.^50^, ^51^, ^52^, ^53^

### Clinical Features and Diagnosis

Presenting maternal symptoms may include abdominal pain localized to the right upper quadrant, nausea, vomiting and fatigue.^54^ Severe kidney injury is generally uncommon in HELLP, although older studies report acute kidney injury in 10% patients with up to 40% of these patients requiring kidney replacement therapy. We speculate that the older studies may not have sufficiently evaluated for aHUS, where renal injury is more common and severe.^55^^,^^56^ Therefore, aHUS/CM-TMA should be considered in the differential in these cases.

The diagnosis of HELLP should be considered once p-TMA is identified. It is diagnosed by the presence of elevated LDH with a level > 600 IU/l, transaminases (AST or ALT) greater than twice the upper limit of normal and thrombocytopenia with platelet count <100 k/ul^57^ (Figure 2). Additional testing is recommended to confirm that LDH elevation in HELLP is due to microangiopathic hemolysis (e.g., haptoglobin, peripheral smear). HELLP is often associated with PE-SF, which is defined as BP greater than 160/110 mm Hg on >2 occasions, and the presence of 1 of the following clinical or laboratory findings: thrombocytopenia with platelet count <100 k/ul, abnormal liver function tests with transaminase levels twice the upper limit of normal or persistent right upper quadrant pain, presence of acute kidney injury, intractable headache, pulmonary edema or visual disturbances.^58^

The recent PRAECIS (Preeclampsia Risk Assessment: Evaluation of Cut-offs to Improve Stratification) study, the largest prospective USA study to date that included 31% Black and 16% Hispanic women, identified and validated an sFlt-1:PlGF ratio to aid in the risk stratification of pregnant women (>18 years of age between 23 weeks 0 days and 34 weeks 6 days’ gestation) hospitalized for hypertensive disorders (preeclampsia, chronic hypertension with or without superimposed preeclampsia, or gestational hypertension) for developing PE-SF^59^ (See Table 1). The investigators sought to identify the sFlt-1:PlGF ratio value that could discriminate between those women likely to develop PE-SF and those who do not. An sFlt-1:PlGF ratio of ≥40 provided an 81% sensitivity (95% confidence interval [CI]: 70–90), 81% specificity (95% CI: 74–87), positive predictive value of 66% (95% CI: 55–76) and negative predictive value of 90% (95% CI: 84–95) for the development of PE-SF within 2 weeks of enrollment in the derivation cohort. Investigators demonstrated that women who developed PE-SF had sFlt-1:PlGF ratios approximately 40 times higher than those who did not (291 [interquartile range, 121–777] vs. 7 [interquartile range, 3–40], respectively; *P* < 0.001). An sFlt-1:PlGF ratio ≥40 also yielded a 94% sensitivity (95% CI: 89–96) and 75% specificity (95% CI: 70–79), as well as positive predictive value of 65% (95% CI: 59–71) and a negative predictive value of 96% (95% CI: 93–98) for developing PE-SF in ensuing 2 weeks in the validation cohort. An sFlt-1:PlGF ratio of 40 or higher was also associated with a higher risk of adverse outcomes related to pregnancy latency and composite maternal morbidity.

The ACOG has published the following clinical practice update based on the PRAECIS study (April 2024)^60^:

1. There are insufficient data to recommend management strategies after a positive or negative test result. The sFlt-1:PlGF ratio alone should not replace current clinical criteria for diagnosing or excluding a diagnosis of PE-SF. If the sFlt-1:PlGF ratio is used for patients who were hospitalized admitted between 23 and 35 weeks of gestation with hypertensive disorders, the test is a complementary risk stratification screen to add to the diagnostic work-up of PE-SF. All patients hospitalized for hypertensive disorders of pregnancy should continue to receive the current recommended approach for preeclampsia workup and management, including serial blood pressure assessment, symptom monitoring, and laboratory surveillance.
2. A positive sFlt-1:PlGF ratio of ≥40 alone is not diagnostic of PE-SF. Conversely, although a negative sFlt-1:PlGF ratio of <40 is reassuring and suggests that the likelihood of developing PE-SF within 2 weeks is <5%. A value <40 can miss cases of PE-SF, and continued close maternal and fetal surveillance is warranted if there are persistent signs or symptoms of preeclampsia.
3. The sFlt-1:PlGF ratio should not be used for patients who did not meet inclusion criteria for the PRAECIS trial (e.g., avoid testing in asymptomatic, nonhospitalized individuals at <23 weeks or >35 weeks of gestation and in postpartum individuals).

### Treatment and Prognosis

Definitive treatment of HELLP syndrome centers around delivery of the fetus and placenta regardless of gestational age (Figure 2). HELLP syndrome should begin to resolve within 3 to 4 days of delivery. Persistence of severe thrombocytopenia (<30,000/μl) or acute kidney injury in the postpartum period beyond 72 hours should prompt consideration of an alternate diagnosis, such as TTP or aHUS/CM-TMA. TTP can be ruled out by checking an ADAMTS13 activity level. If there is a delay in the turnaround time for this diagnostic test, empiric treatment with plasmapheresis should be initiated while awaiting results, especially if profound thrombocytopenia or neurologic symptoms are present. A recent study demonstrated that HELLP can potentially be differentiated from CM-TMA by the combination of serum creatinine and LDH^61^ (See Table 1). Although the importance of LDH in differentiating TMAs has been reported before,^62^ this study included a direct comparison of 46 cases of pregnancy-associated aHUS and 45 cases HELLP syndrome, with detailed analysis of laboratory parameters in the postpartum period. Receiver operating curve analysis revealed that serum creatinine (0.996 [95% CI: 0.99–1.0]) and LDH levels (0.91 [95% CI: 0.83–0.98]) had a strong positive correlation with the diagnosis of aHUS. Youden J statistic (sensitivity + specificity − 1) was used to establish the optimal cutoff points for serum creatinine and LDH to differentiate aHUS from HELLP syndrome in the postpartum period. The authors reported that postpartum serum creatinine levels were ≥1.9 mg/dl in 98% of aHUS cases compared with 2% of HELLP cases (*P* < 0.001), and serum LDH levels were ≥1832 U/l in 77% of aHUS cases compared with 0% of HELLP cases (*P* < 0.001). When evaluated together, the combination of serum creatinine ≥1.9 mg/dl and LDH ≥600 U/l showed the greatest utility for diagnosis of aHUS in the study population (specificity 100%, sensitivity 97%, J statistic 0.97), as it was seen in 91% of aHUS cases compared with 0% of HELLP cases (*P* < 0.001). The authors acknowledged that the study may have been limited by publication bias because of publication of more severe postpartum aHUS cases in the literature and therefore, it is possible that even lower thresholds for creatinine and LDH may be useful if milder cases of aHUS were included. Although the study requires further validation, until more advanced testing modalities are available, serum creatinine and LDH may be used to quickly differentiate HELLP from aHUS/CM-TMA.

Individuals who experience HELLP syndrome are at an increased risk for development of preeclampsia and HELLP during subsequent pregnancies and for the development of chronic hypertension, seizures, stroke, cardiovascular disease, pulmonary edema, kidney failure and depression.^63^ Fetal complications can include intrauterine growth restriction and prematurity.

### CM-TMA

CM-TMA results from an overactivation of the alternative pathway (AP) of complement (Figure 1c). Incidence of CM-TMA is noted to be around 1 to 2 cases per million population for adults aged ≥18 years.^64^ Pregnancy related CM-TMA is reported to occur in 1 in 25,000 pregnancies accounting for 7% of all TMA cases with 79% of these presenting in the postpartum period.^65^, ^66^, ^67^

### Etiology/Pathophysiology

The AP has the capacity to deposit on a pathogen without need for prior contact/exposure. This innate immune, self-amplifying defense mechanism results from the lability of an internal thioester bond. This leads to generation of small amount of autoactivated C3 continuously generated in blood at 1% to 2%/h (called tick-over).^68^ If it deposits on healthy self-tissue or ticks over in the fluid phase, host regulatory proteins immediately inactivate it. However, if it is deposited on a microbe, activated C3 can be rapidly amplified by engaging 2 proteases, Factor B and Factor D, along with a stabilizing protein, properdin, to create the powerful AP C3 convertase.^68^ Contained within the AP is an efficient feedback or amplification loop for the generation of large amounts of C3b to opsonize a pathogen. Because the complement system provides a rapidly activated and potent surveillance mechanism for the host, strict control is required to avoid damage to self. Thus, inhibition of complement activation is mediated by host regulators in the plasma as well as on cells. A normal complement system is finely tuned and prepared to properly deal with injury. In CM-TMA, an excessive degree of AP activation takes place in the setting of dysfunctional control, leading to microthrombi, especially in the glomeruli of the vulnerable kidney.^68^ The most common etiology of a dysregulated complement system in CM-TMA is a heterozygous, loss-of-function mutation in a regulator of the AP (such as in factor H [FH], factor I [FI] or membrane cofactor protein [MCP, CD46]) that leads to haplo-insufficiency as identified in 60% to 70% patients^68^ (Figure 1c). In these cases, the protein is generally (i) not synthesized, (ii) synthesized but not secreted, or (iii) secreted in normal amounts but is dysfunctional.^68^ Less frequently, a gain-of-function mutation in a complement activator (C3, factor B) is identified.^69^ Acquired deficiencies in the form of autoantibodies against FH also occur in ∼5% to 10% of patients.^70^ A genetic defect, namely a homozygous deletion in CFHR1 and CFHR3 (complement FH-related 1 and 3) may predispose to the development of FH autoantibodies.^71^ Disease penetrance is ∼50%, suggesting that an environmental trigger is necessary in most cases. The penetrance may increase significantly, however, if the patient carries more than 1 disease-associated variant or a risk polymorphism as further described below.^72^ Pregnancy (particularly the postpartum period) is considered 1 of the major triggers for CM-TMA^73^ Pregnancy is an immunologically privileged condition where placental damage is prevented by regulators of complement activation, such as, decay accelerating factor (DAF, CD55), MCP and CD59. These regulators assist in the clearance of placental fragments that enter the maternal circulation as a result of syncytiotrophoblast turn-over. Furthermore, levels of many complement proteins increase during pregnancy. Reversal of these phenomena in the postpartum period is speculated to predispose to development of CM-TMA.^74^ Complement activation can also occur due to an imbalance in angiogenic factors, already described above, under the pathophysiology of HELLP.

### Clinical Features and Diagnosis

Presenting symptoms can vary widely and consist of nausea, vomiting, abdominal pain, headache, altered mental status, and hypertension.^1^ Renal impairment is almost always seen with CM-TMA and often more severe than TTP.^61^ Markedly elevated creatinine and LDH strongly suggests pregnancy associated CM-TMA and can be used to differentiate from HELLP in the postpartum period.^61^ A serum creatinine of ≥1.9 mg/dl or an LDH ≥1832 U/l or a serum creatinine ≥ 1.9 mg/dl + LDH ≥ 600 U/l has been reported to be >95% specific for a diagnosis of CM-TMA.^61^ Evaluation for underlying genetic variants in complement proteins and FH autoantibodies should be conducted in all patients^4^ (Figure 2). The clinically validated next-generation sequencing-based complement disease panel currently consists of 15 genes (ADAMTS13, C3, CD46, CFB, CFH, CFHR1, CFHR2, CFHR3, CFHR4, CFHR5, CFI, DGKE, THBD, MMACHC, and PLG).^75^ If identified, interpretation of variants may require additional functional assays, biomarker testing, or in select cases need for recombinant protein production followed by structure-function assessment of the variants.^75^^,^^76^ Genetic and functional testing can take up to 4 to 6 weeks, therefore are not used to make a diagnosis of CM-TMA but are important to determine risk of relapse and recurrence of disease as well as to establish duration of treatment.^75^^,^^76^ Common polymorphisms in complement genes, have also been associated with increased risk of developing CM-TMA.^77^ It is speculated that these inherited common polymorphisms may affect the delicate balance between activation and regulation and thereby ‘set’ an individual’s intrinsic complement activity and susceptibility to AP-driven disease; this repertoire has been referred to as the complotype. However, because of lack of clear data on the extent to which they affect disease susceptibility, these variants are often not included in clinical genetic reports.

### Treatment and Prognosis

Prior to the approval of C5 inhibitors, treatment with PLEX was the mainstay for the management of CM-TMA although the overall efficacy was poor^4^ (Figure 2). The rationale behind this treatment is to reduce the quantity of a mutant protein by plasmapheresis and then deliver a functionally normal protein by plasma infusion. Plasma therapies still remain the initial treatment of choice in countries where complement inhibitors are not available, or the cost precludes its use.^4^

Eculizumab is a humanized monoclonal antibody to C5 which blocks activation of the terminal pathway and prevents membrane attack complex (MAC) formation.^78^ Multiple studies and case reports have now shown relative efficacy and safety of eculizumab in pregnancy and breast feeding and improved outcomes including, decreased progression to ESKD, less time on dialysis, and successful remission of disease.^4^^,^^79^^,^^80^ When used in pregnancy, eculizumab has been detected at low concentrations in umbilical cord blood samples, suggesting that it crosses placenta at low levels.^80^ Treatment with eculizumab during pregnancy does not seem to alter the complement system activity of the newborn.^81^ It is also considered safe with lactation as it has not been detected in breast milk samples among women receiving eculizumab.^80^

A newer longer acting C5 inhibitor, ravulizumab is also approved for CM-TMA.^82^ Ravulizumab has been engineered from eculizumab by changing 4 amino acids. This change preserves the binding of ravulizumab to C5 in serum but allows it to dissociate from C5 in the acidified endosome (pH 6.0).^82^ Additionally, these amino acid alterations also result in an increased efficiency of neonatal Fc receptor-mediated recycling of ravulizumab; thereby, leading to an increased half-life of ∼52 days compared to ∼11 days for eculizumab.^83^ Ravulizumab is not recommended in pregnancy or lactation due to the lack of safety data. Due to the structural modifications noted above, ravulizumab has more than 10-times greater affinity for the neonatal Fc receptor compared to eculizumab, enabling higher uptake into the fetal circulation and breastmilk. However, its use in postpartum CM-TMA has been reported in a subgroup analysis of 8 patients from a phase 3 multicenter trial of treatment-naïve adult patients with aHUS^82^ (See Table 1). All patients presented with acute severe medical emergency associated with pregnancy or delivery (2 with preeclampsia, 1 with gestational diabetes, 2 with placental abruption and 5 were on dialysis). Complete TMA response (defined as LDH normalization, platelet count normalization and ≥25% improvement in serum creatinine from baseline) was observed in 7 of 8 patients in 31 days and all patients on dialysis came off dialysis within 21 days. The use of ravulizumab in pregnancy will need to be confirmed in studies with larger sample sizes. The predominant concern with using anticomplement therapy is a life-threatening infection with *N meningitidis* (because of blockage of the terminal complement pathway). Meningococcal vaccination must be administered to everyone undergoing treatment with eculizumab or ravulizumab.^84^ In addition, when eculizumab or ravulizumab must be given urgently, appropriate antibiotics should be used for at least 14 days after the first dose of meningococcal vaccine.^84^

Several other drugs for CM-TMA are also in development or undergoing clinical trials. These novel complement inhibitors include crovalimab (anti-C5), nomacopan (anti-C5), pegcetacoplan (anti-C3) and iptacopan (anti-factor B), and narsoplimab (mannose-binding lectin-associated serine protease 2 [MASP-2]).^85^ Prospective, randomized controlled trials are required to investigate the use of these therapies in CM-TMA and particularly their efficacy and safety in pregnancy associated CM-TMA.

There is no established best treatment for FH autoantibodies. A combination of many different therapies has been used and reported over the years in nonpregnant patients. These include PLEX, eculizumab, prednisone, rituximab, and cyclophosphamide. In our experience, none of these therapies by themselves have been effective in removing FH autoantibodies.^86^^,^^87^ A combination of PLEX and steroids has sometimes helped with decreasing autoAb titres.^88^ Also, in a subset of kidney transplant patients, the autoantibody titers declined after being on maintenance immunosuppression for 6 to 24 months.^89^^,^^90^ For pregnancy and postpartum, however, the options are more limited.

The optimal duration of anticomplement therapy also remains unclear. During the Kidney Disease Improving Global Outcomes Consensus Conference held in 2015, experts recommended that discontinuation can be considered on a case-by-case basis in patients after at least 6 to 12 months of treatment and at least 3 months of stabilization of the kidney function. Since then, a few other studies have shown that stopping anticomplement therapy is feasible in a carefully selected subset of patients.^91^ These patients include those who have responded promptly to treatment, with recovery of kidney function back to baseline and if no underlying genetic etiology is identified. For those in whom a genetic variant of uncertain significance is identified, a sequential and systematic analysis of clinical history, antigenic, genetic, functional and biomarker analyses should be undertaken for individualized decision making. After stopping therapy, close monitoring, and prompt reinitiation of treatment is critical, if relapse occurs. Ultimately, the decision to stop treatment should involve shared decision making with patients after understanding all the risks and benefits.

Maternal complications of pregnancy associated CM-TMA include increased risk of preterm delivery, disseminated intravascular coagulation, ESKD, stroke, and even death.^77^^,^^92^, ^93^, ^94^ Babies born to mothers with CM-TMA are often small for gestational age and have low birth weight with increased risk for fetal morbidity and mortality.^66^^,^^95^

### APS associated TMA

APS is a systemic autoimmune disorder characterized by development of antiphospholipid antibodies (aPLAb) which include lupus anticoagulant, anticardiolipin antibodies and anti-beta2 glycoprotein I.^96^ The estimated prevalence of APS ranges from 40 to 50 patients per 100,000.^97^^,^^98^ It is estimated that aPLAbs are associated with 50,000 pregnancy losses, 110,000 strokes, 100,000 myocardial infarctions, and 30,000 deep vein thrombosis every year in the US.^99^ APS can occur as a primary condition in ∼50% patients or in the setting of systemic lupus erythematosus or another systemic autoimmune disease.^100^ TMA can be associated with APS and has an incidence of about 8% to 31% in primary cases.^101^ Patients with APS and TMA have an increased risk of stroke and arterial thrombotic events compared with APS without TMA.^101^

### Etiology/Pathophysiology

Antiphospholipid antibodies can lead to thrombosis by inhibition of the anticoagulant cascade and fibrinolytic activity and enhancing platelet aggregation as well as complement activation^96^ (Figure 1d). The current model of aPLAb-mediated thrombosis however is better defined by a “two-hit” hypothesis since thrombosis alone is often not sufficient to explain the placental damage observed in APS.^95^ Patients with positive aPL antibodies (first hit) often need an additional inciting agent or trigger (second hit) to manifest disease. Triggers can include infection, sepsis, surgery, smoking, or hormonal changes during pregnancy.^102^ Overall, these mechanisms result in a proinflammatory state involving activation of neutrophils and monocytes, disruption of the phospholipid bilayer and complement activation.^96^ Animal models have demonstrated that inflammatory processes involving complement activation contribute most to poor obstetric outcomes by promoting coagulation and cellular injury. Additionally, the interaction of aPLAb with the disrupted phospholipid bilayer can lead trophoblast dysfunction and decidual necrosis.^76^^,^^103^, ^104^, ^105^, ^106^ Neutrophil extracellular traps (NETs) have also been implicated in the pathogenesis of APS and may aggravate thrombosis through activation of the coagulation cascade and inhibition of anticoagulant factors. Antiphospholipid antibodies themselves promote NET formation. Moreover, NETs formed in APS appear resistant to degradation. Anti-NET antibodies (particularly IgM), which may act by stabilizing NETs, are also markedly increased in patients with APS.^107^

### Clinical Features and Diagnosis

APS may involve virtually all organ systems, resulting in stroke, skin ulcerations, nephropathy, seizures, and cognitive decline.^108^ The clinical manifestations can be classified as thrombotic, obstetric or catastrophic. Thrombotic APS is mainly characterized by venous, arterial and small vessel thrombotic events in different organs.^108^ A hallmark of obstetric APS is pregnancy complications, which include preeclampsia, recurrent early pregnancy loss, fetal death or premature birth due to intrauterine growth restriction.^109^ Lupus anticoagulant positivity has been shown in several prospective studies to be a strong predictor of poor pregnancy outcomes.^110^^,^^111^ Patients who carry all 3 autoantibodies (triple aPL positivity) are reported to have significantly worse pregnancy outcomes with increased risk of fetal loss.^109^ Approximately 1% of patients with APS may develop catastrophic APS (CAPS) which is a severe and life-threatening form characterized by microvascular and macrovascular thrombosis that develop simultaneously or over a short period of time leading to multiorgan failure and a significantly increased risk of mortality.^96^

Renal involvement in APS is characterized by findings consistent with renal artery or vein thrombosis, TMA on biopsy (most frequently seen with catastrophic APS), membranous glomerulonephritis, focal segmental glomerulosclerosis, focal cortical atrophy with interstitial fibrosis secondary to tissue ischemia.^112^ Patients often present with severe hypertension and proteinuria which can range from mild to nephrotic range.^112^^,^^113^ TMA associated with APS is more commonly renal-limited and may lack the systemic hematologic findings of anemia, thrombocytopenia or presence of schistocytes.^113^

Diagnosis should be suspected when patients present with thrombotic events and adverse pregnancy outcomes especially in the setting of recurrent miscarriages and/or systemic lupus erythematosus.^108^ A revised APS classification criteria has recently been published by the American College of Rheumatology and European League Against Rheumatism (Figure 3).^114^ Laboratory evaluation should include complete blood count, renal function panel, coagulation profile, urinalysis and urine protein evaluation, antinuclear Ab, antidouble stranded DNA Ab and aPLAb.^108^ aPL antibodies are usually obtained at time of event and then again ≥12 weeks later to confirm the diagnosis.^108^

**Figure 3:**
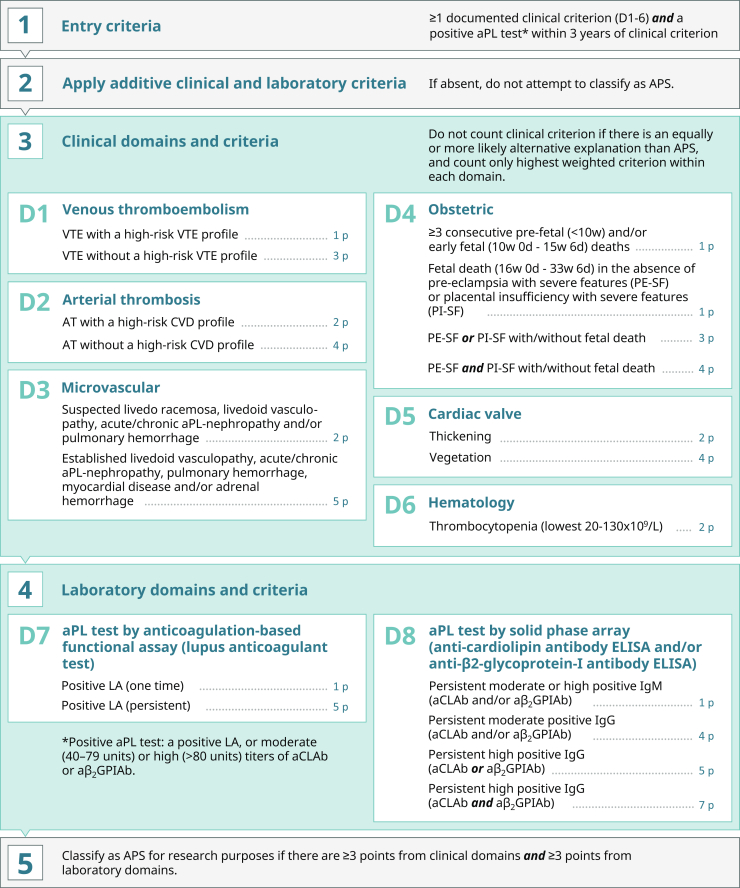
Revised American College of Rheumatology (ACR)/European League Against Rheumatism (EULAR) classification criteria for the diagnosis of APS. APS, antiphospholipid syndrome; aPL, antiphosopholipid; aβ_2_GPIAb, anti-β2-glycoprotein-I antibody; aCLAb, anticardiolipin antibody; AT, arterial thrombosis; CVD, cardiovascular disease; D1-D8, domains; ELISA, enzyme-linked immunosorbent assay; LA, lupus anticoagulant; VTE, venous thromboembolism.

### Treatment and Prognosis

Warfarin (or acenocoumarol) is the preferred anticoagulant for nonpregnant patients presenting with their first thrombotic event as well as for secondary prevention.^115^ International Normalized Ratio (INR) goal is usually between 2 and 3^116^^,^^117^ (Figure 2). Aspirin is added to warfarin if there is also a history of arterial thrombosis.^118^^,^^119^ In pregnancy, however, low molecular weight heparin (LMWH) is used in place of warfarin due to its known teratogenicity.^120^, ^121^, ^122^ If there is a history of former thrombotic event, expert opinion recommends LMWH along with low dose aspirin due to the prothrombotic nature of pregnancy.^120^, ^121^, ^122^ In patients with no prior history of thrombosis but with laboratory criteria for aPL and ≥1 fetal loss at ≥10 weeks of gestation or ≥3 unexplained consecutive, spontaneous pregnancy losses at <10 weeks of gestation treatment also consists of low dose aspirin and prophylactic LMWH. If a patient has positive aPL antibodies and ≥1 preterm delivery before 34 weeks of gestation or other findings consistent with placental insufficiency without prior thrombotic event, patient is treated with low dose aspirin or LMWH alone.^120^, ^121^, ^122^ IVIG is reserved for those with a history of pregnancy losses despite appropriate anticoagulation.^118^ The management of catastrophic APS is similar, and patients are treated with anticoagulation, high dose solumedrol, and PLEX or IVIG.^123^ If refractory to treatment, a trial of rituximab or eculizumab can be considered.^115^^,^^124^

Statins upregulate endothelial nitric oxide synthase and may be protective in APS although they are not widely used in pregnancy.^125^ Hydroxychloroquine can also be considered for those with APS secondary to systemic lupus erythematosus.^126^ Direct oral anticoagulants are not used since they have been associated with increased risk of thrombotic events and strokes in several studies.^116^^,^^117^

In a 2019 study, 18 women with aPLAb-related obstetric complaints who failed to achieve live births despite standard therapy (LMWH plus low dose aspirin plus hydroxychloroquin) were treated with TNF-α blockers.^127^ Sixteen patients were started on adalimumab and 2 on certolizumab. Twelve of 18 women completed gestation with no maternal-fetal adverse effects, however, miscarriage or implantation failure recurred in 6 patients. Limitations of the study include small sample size and pregnancy achieved through in vitro fertilization techniques, thereby making it easier to time administration of TNF-a blockers compared to those planning natural pregnancies. Moreover, cases with arterial or venous thrombosis were excluded. Therefore, these studies need to be interpreted with caution and there is need for ongoing research and well-designed trials (See Table 1).

Long term outcomes for pregnant patients with APS can vary, however, the overall rate of live birth is lower (∼73%) compared to the general population (∼90%).^16^^,^^128^ The most common obstetric complication is recurrent miscarriage which occurs in about 1% of the general population as compared to 10% to 15% of women with APS.^129^^,^^130^ High titer anticardiolipin and prior fetal loss carry an 80% risk of future pregnancy loss.^131^ After pregnancy, women with APS remain at higher risk of venous thromboembolism and ischemic cerebrovascular disease.^132^ Children born to mothers with APS may also have higher rates of learning disabilities although these studies have been small and conclusive evidence of this association remains to be seen.^133^^,^^134^

### Conclusion

p-TMA remains a challenging diagnosis given several overlapping features between the 4 most common etiologies. A thorough clinical history, pertinent laboratory data, and early identification of patterns of injury can serve to clarify the diagnosis in most cases. The mainstay of TTP treatment in pregnancy centers around steroids and/or PLEX with fresh frozen plasma. However, novel therapies are coming soon as outlined above and summarized in the what’s new section (Table 1). For HELLP and preeclampsia, the only current treatment is delivery of the baby. Therefore, opportunities for future research include improved predictive models, better defining the pathogenesis of preeclampsia, elucidating immunologic predispositions and genetic etiologies. C5-inhibitors are an effective treatment for CM-TMA in pregnancy and the postpartum period. Many new anticomplement drugs are in trials and their safety and efficacy in pregnancy will need to be determined. Areas of further research in CM-TMA include establishing an effective treatment for FH-autoantibodies, defining the duration of anticomplement therapy (i.e., who needs to be treated and for how long) as well elucidating the role of common complement polymorphisms in CM-TMA. Finally, APS is another devastating condition associated with pregnancy and should be considered in the differential diagnosis of pregnancy-associated TMAs. Our understanding of the clinical and laboratory features of APS and the optimal treatment of APS in pregnancy is still evolving.

## Disclosure

AJ serves on the scientific advisory boards of Alexion, AstraZeneca Rare Disease, and Novartis International AG, and serves as a consultant for Dianthus Therapeutics and Aurinia Pharmaceuticals. She is also a Principal Investigator for Apellis Pharmaceuticals and Novartis International AG and receives royalty from UptoDate. RB serves on the scientific advisory board and speakers bureau for Alexion, AstraZeneca Rare Disease, and serves on advisory boards for UCB Biosciences, Comanche Biopharma and Roche Diagnostics.
